# Draft Genome Sequences of Photobacterium damselae subsp. piscicida SNW-8.1 and PP3, Two Fish-Isolated Strains Containing a Type III Secretion System

**DOI:** 10.1128/MRA.00426-19

**Published:** 2019-05-23

**Authors:** Saqr Abushattal, Ana Vences, Nuno M. S. dos Santos, Ana do Vale, Carlos R. Osorio

**Affiliations:** a Departamento de Microbioloxía e Parasitoloxía, Instituto de Acuicultura, Universidade de Santiago de Compostela, Santiago de Compostela, Spain; b Fish Immunology and Vaccinology Group, IBMC-Instituto de Biologia Molecular e Celular, Universidade do Porto, Porto, Portugal; c i3S-Instituto de Investigação e Inovação em Saúde, Universidade do Porto, Porto, Portugal; Loyola University Chicago

## Abstract

Here, we report the draft genome sequences of two strains of the fish pathogen Photobacterium damselae subsp. piscicida, isolated from Salmo salar (SNW-8.1) and Seriola quinqueradiata (PP3). The identification of a type III secretion system in the two genomes furthers our understanding of the pathobiology of this subspecies.

## ANNOUNCEMENT

Photobacterium damselae subsp. piscicida, a marine bacterium of the family *Vibrionaceae*, is one of the most devastating bacterial pathogens in marine fish aquaculture (1). Demonstrated virulence factors in this subspecies include the apoptosis-inducing toxin AIP56 encoded within plasmid pPHDP10 (2) and the siderophore piscibactin system encoded within plasmid pPHDP70 (3). However, little is known about additional potential virulence factors. Here, we report that strains SNW-8.1 and PP3 carry genes of a type III secretion system (T3SS), which suggests that this virulence factor might constitute a widespread trait within the subspecies.

Strain SNW-8.1 was isolated from diseased salmon (Salmo salar) in an aquaculture site in Spain, and PP3 was isolated from diseased yellowtail (Seriola quinqueradiata) in Japan. Isolates were cultured at 25°C on Trypticase soy agar (TSA) supplemented with 1% NaCl. The two isolates were identified as P. damselae subsp. *piscicida* on the basis of their 16S rRNA gene sequences (4) and phenotypical traits as previously described (5). High-purity genomic DNA was extracted from the two isolates using the GNOME DNA kit (MP Biomedicals), following the manufacturer’s recommendations. The library for SNW-8.1 was prepared using the TruSeq DNA PCR-free kit (Illumina) and sequenced with the Illumina HiSeq 2000 platform (2 × 100-bp paired-end reads), generating 5.7 million reads. For the two genome sequences, the quality of the raw data was checked using FASTQC tools (https://www.bioinformatics.babraham.ac.uk/projects/fastqc/). Raw reads of SNW-8.1 were trimmed using Trimmomatic v0.39 (6). The reads were assembled with SPAdes 3.6 using default settings (7). The assembly of SNW-8.1 consisted of 641 contigs (>200 bp) with an *N*_50_ value of 13,314 bp, totaling 4,041,749 bp with a G+C content of 40.8%. Coverage was calculated as 298×. For sequencing library preparation of strain PP3, genomic DNA was mechanically sheared in a ultrasonicator (Covaris), ends were enzymatically repaired, and adaptors (Illumina) were ligated. The library was sequenced using the Illumina MiSeq platform (2 × 150-bp paired-end reads). This sequencing generated 2.86 million reads, and the data were assembled with MEGAHIT v1.0 using default settings (8). The assembly of PP3 consisted of 520 contigs (>200 bp) with an *N*_50_ value of 13,455 bp, totaling 4,120,359 bp with a G+C content of 40.8%. Coverage for PP3 was calculated as 208×. As previously reported (9), P. damselae subsp. *piscicida* genomes contain a large number of repetitive insertion sequence (IS) elements that explain the large number of contigs yielded after genome sequence assembly.

The two assembled draft genomes were annotated using the NCBI Prokaryotic Genome Annotation Pipeline (10). Annotation revealed that the two strains contained sequences homologous to the virulence plasmids pPHDP10 (encoding the AIP56 toxin) and pPHDP70 (encoding the siderophore piscibactin iron sequestering system). Notably, the two strains possessed putative genes for the T3SS, a virulence factor that has been rarely reported in this fish-pathogenic bacterium so far. Indeed, two draft genomes of strains isolated in Spain lacked such a secretion system (9). The T3SS inner membrane channel EscV encoded within SNW-8.1 (locus tag E4T25_07185) and PP3 (locus tag E4T26_05130) genomes was 99% to 100% identical to EscV proteins annotated in the genome of P. damselae subsp*. piscicida* OT-51443 (11) and in the pPHDD203 plasmid of the type strain CIP102761 of P. damselae subsp. damselae (12), as revealed by BLAST search (13). Similarly, SNW-8.1 and PP3 draft genomes encoded putative T3SS YopR effector proteins (locus tags E4T25_08745 and E4T26_17660, respectively), which showed >98% identity to YopR proteins of P. damselae subsp. *piscicida* OT-51443 and P. damselae subsp. damselae CIP102761. The presence of type III secretion system genes, in addition to the virulence plasmids pPHDP10 and pPHDP70, underscores the high pathogenic potential of P. damselae subsp. *piscicida* SNW-8.1 and PP3. The draft genomes reported here will be useful for examining whether the type III secretion system is widespread in other isolates of Photobacterium damselae subsp. *piscicida* throughout the world.

### Data availability.

These two draft genome projects have been deposited at GenBank/EMBL/DDBJ under the GenBank accession number SRHD00000000, BioProject accession number PRJNA529569, and BioSample accession number SAMN11269579 for SNW-8.1 and under the GenBank accession number SRHT00000000, BioProject accession number PRJNA529570, and BioSample accession number SAMN11269591 for PP3. The raw reads were deposited in the SRA under accession numbers SRR8902871 (strain SNW-8.1) and SRR8954861 (strain PP3).
